# A Rare Case of Pseudomonas aeruginosa Keratoscleritis: Highlighting Early Diagnosis and Aggressive Treatment

**DOI:** 10.7759/cureus.92228

**Published:** 2025-09-13

**Authors:** Sally Al Hassan, Jana Tabaja, Randa Haddad, Joanna S Saade

**Affiliations:** 1 Ophthalmology, American University of Beirut Medical Center, Beirut, LBN; 2 Cornea and Refractive Surgery, American University of Beirut Medical Center, Beirut, LBN

**Keywords:** corneal epithelial defect, corneal ulcer, infectious scleritis, keratoscleritis, pseudomonas aeruginosa

## Abstract

Background: *Pseudomonas aeruginosa* keratoscleritis (PAK) is an uncommon but aggressive ocular infection that can rapidly threaten vision if not recognized and treated promptly.

Case presentation: A 75‑year‑old woman presented with a large corneal epithelial defect, stromal infiltrates, and hypopyon. She was initially managed empirically for fungal keratitis but later diagnosed with PAK following scleral nodule excision and positive cultures for *Pseudomonas aeruginosa *(*P. aeruginosa*).

Management and outcome: Intensive therapy, including topical and systemic antibiotics, subconjunctival injections, and autologous serum tears, gradually improved corneal ulceration and scleritis. Although her visual acuity remained severely impaired, early initiation of targeted antimicrobial therapy preserved ocular integrity and avoided surgical intervention.

Conclusion: PAK is a rare, sight-threatening infection with high morbidity if treatment is delayed. This case highlights the importance of early and accurate diagnosis, together with prompt and aggressive antimicrobial management to prevent complications and preserve the globe.

## Introduction

Scleritis is regarded as an infrequent yet severe medical condition that necessitates prompt medical intervention due to the risk of diminished vision or complete vision loss in the impacted eye(s) [1]. Infectious scleritis, whether occurring on its own or alongside keratitis, presents significant challenges in treatment and management [2]. *Pseudomonas aeruginosa (P. aeruginosa*) remains the most prevalent cause of bacterial keratoscleritis, responsible for 51-81% of these cases [3].

The aggressive course of *Pseudomonas aeruginosa *keratoscleritis (PAK) relates to its unique virulence factors, including proteases, exotoxins, biofilm formation, and a type III secretion system, all of which contribute to corneal and scleral destruction [4-7]. Despite recognition of these mechanisms, the clinical literature remains sparse, with most publications limited to small case series. This report adds to existing knowledge by describing a patient with advanced PAK who presented early and was successfully managed with intensive antimicrobial therapy, including systemic, topical, and subconjunctival antibiotics supplemented by autologous serum tears. Unlike many published cases, our patient required no surgical intervention, underscoring how timely diagnosis and targeted antimicrobial therapy can preserve ocular integrity even in severe presentations.

## Case presentation

**Figure 1:**
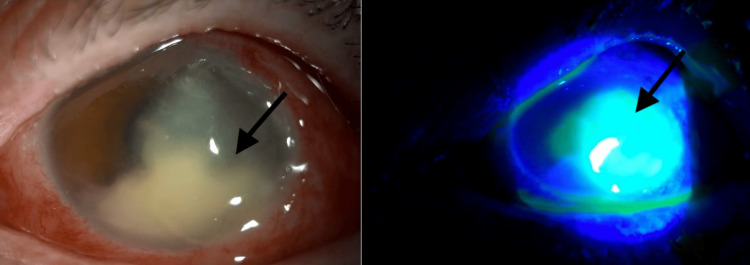
Slit-lamp image at presentation with and without fluorescein Slit-lamp photograph of the left eye at initial presentation showing extensive conjunctival injection, a large epithelial defect over the temporal cornea, dense stromal infiltrates, and a hypopyon occupying 30% of the anterior chamber

A 75-year-old woman presented to the ophthalmology clinic at the American University of Beirut Medical Center after her family noted a whitish deposit over her left cornea that appeared four days prior to presentation. She had seen another ophthalmologist who started her empirically on oral fluconazole 100 mg daily without any topical medication after suspecting a fungal corneal ulcer. No sample was taken for culture and sensitivity at that time. She denied any history of trauma, contact lens wear, or recent surgery. She came to seek a second opinion. Her past medical history was positive for hypertension. On examination, her visual acuity, measured using the Snellen chart, was counting fingers (CF) at 0.5 meters in the right eye (Logmar = 2.3) and light perception in the left eye (Logmar = 3). Slit-lamp examination of the left eye revealed 3+ injection in the conjunctiva. The cornea had a large epithelial defect covering most of the temporal half of the cornea with associated thinning between four and five o’clock close to the limbus. Dense infiltrates were present below the defect. An uneven hypopyon was noted occupying 30 % of the anterior chamber with an extension over the endothelium temporally below the corneal defect (Figure 1). The patient was pseudophakic in this eye with a posterior chamber intraocular lens. B-scan of the left eye revealed a clear vitreous.

At the initial visit, corneal scrapings were obtained for bacterial and fungal cultures, cytology, and smear examination. All tests, including cytology and smear, yielded negative results. Empiric therapy for suspected bacterial keratitis was initiated with fortified amikacin (50 mg/ml) and vancomycin (50 mg/ml) eye drops, administered alternately every 30 minutes in the left eye.

**Figure 2:**
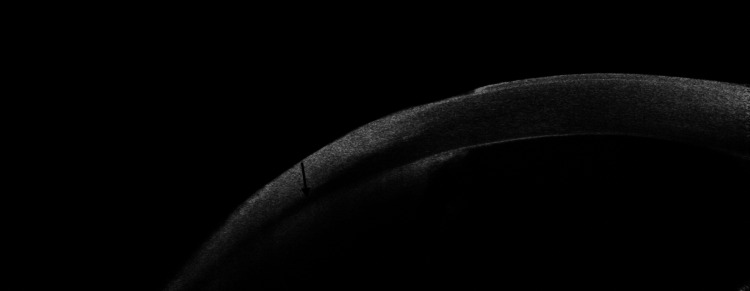
Anterior segment optical coherence tomography (AS-OCT) imaging on day 5 of presentation Anterior segment optical coherence tomography (AS-OCT) image of the left cornea on day 5 of treatment, demonstrating significant corneal thinning indicative of corneal melting

**Figure 3:**
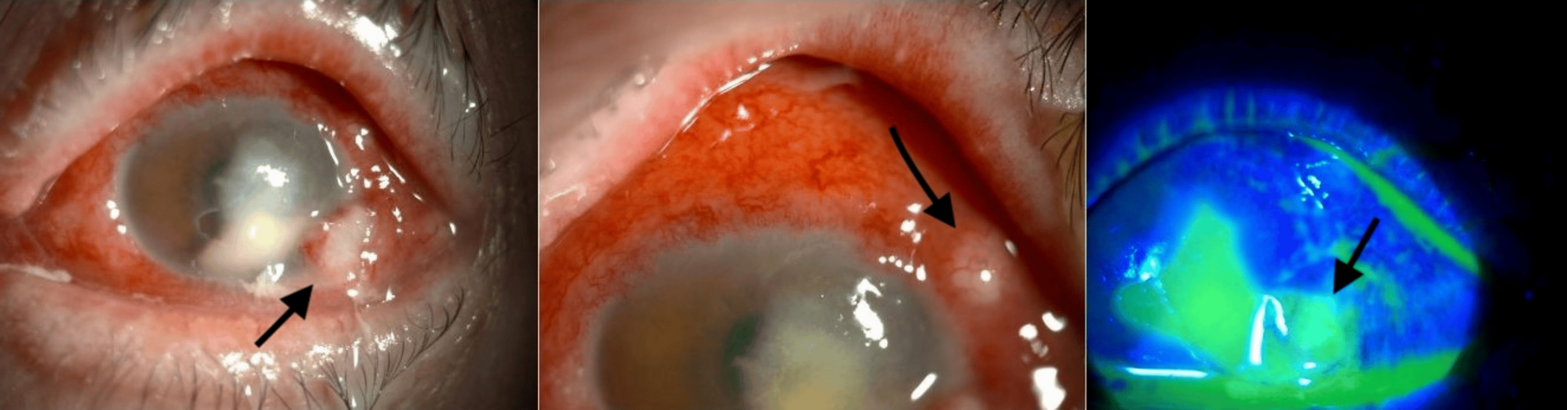
Slit-lamp exam on day 5 of presentation showing scleral nodules Slit-lamp photograph on day 5 of treatment, revealing hard nodules in the sclera that were initially misidentified as conjunctival cysts

The corneal ulcer was closely monitored with repeated clinic visits. By the fourth day, the patient reported decreased eye pain. Slit-lamp examination showed signs of improvement: resolving superior infiltrates and a slightly reduced epithelial defect. However, the appearance of superficial and deep neovessels was temporally noted. A small hyphema developed within the unchanging hypopyon. The most notable finding was the appearance of two conjunctival cysts and inferior pseudomembranes. These were initially thought to be due to the fortified eye drops; therefore, their frequency was decreased. By the fifth day, cultures were still negative. Azithromycin eye drops (1%) were added; 1 drop administered every two hours, in addition to oral doxycycline 100 mg taken daily to help with corneal melting (83 μm, Figure 2). At this point, what was thought to be conjunctival cysts was actually three hard nodules in the sclera (Figure 3). The largest nodule was excised, and samples were sent to pathology and to culturing, looking for a bacterial and/or fungal pathogen. Forty-eight hours later, the culture plates grew *P. aeruginosa*, confirming a diagnosis of PAK. The patient was admitted for intravenous antibiotics and continued eye drops for dual coverage against* P. aeruginosa*. The patient received intravenous ceftazidime and amikacin, supplemented with hourly fortified topical drops (50 mg/ml) and autologous serum tears (AST) every two hours. Subconjunctival amikacin injections were administered on days 2 and 3. Following a negative repeat fungal culture, fluconazole was discontinued. She was discharged on day 4 with oral ciprofloxacin (750 mg twice daily for two weeks), hourly topical antibiotics while awake, and AST four times daily (Table 1).

**Table 1:** Treatment timeline in Pseudomonas aeruginosa keratoscleritis AST: autologous serum tears The table summarizes the stepwise management, including systemic, topical, and subconjunctival antibiotics, culture results, and discharge medications during the patient’s admission

Day	Intervention	Details
1	Initiation of antibiotics	Intravenous ceftazidime and amikacin; hourly topical ceftazidime and amikacin (50 mg/ml); AST every 2 hours
2	Subconjunctival injection	Amikacin 0.3 ml (500 mg/2 ml)
3	Subconjunctival injection	Second amikacin 0.3 ml (500 mg/2 ml)
3	Culture review	Repeat fungal culture negative → fluconazole discontinued
4	Discharge	Oral ciprofloxacin 750 mg twice daily for 2 weeks; hourly topical antibiotics while awake; AST four times daily

**Figure 4:**
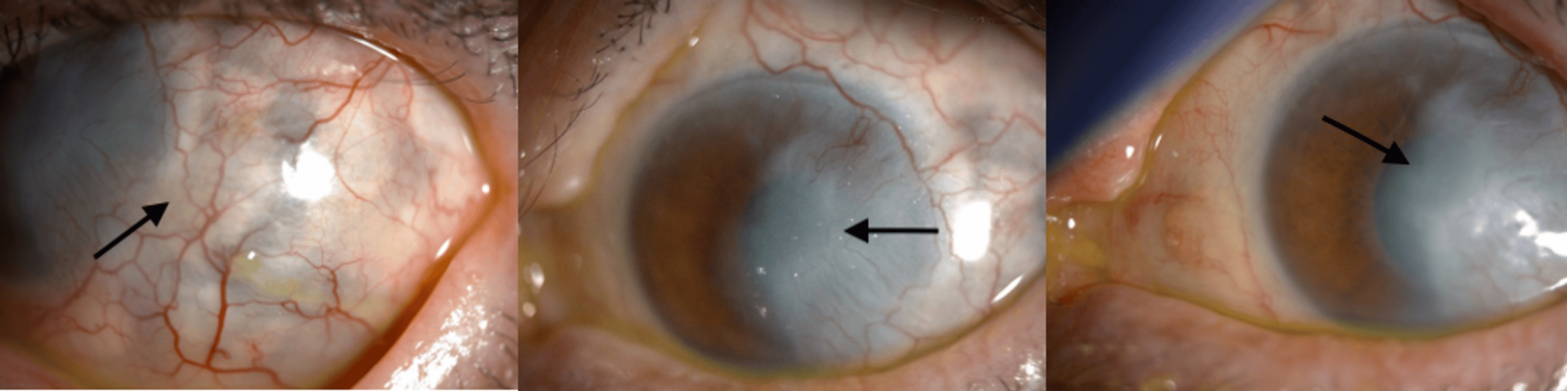
Slit-lamp exam at two-month follow-up Slit-lamp photograph taken two months post-treatment, showing a healed corneal epithelial defect replaced by scarring, with no signs of perforation

Two weeks following discharge, the patient's corneal ulcer had improved. Slit-lamp examination showed a healing epithelial defect, a decrease in the corneal infiltrates, and resolution of the hypopyon. The temporal cornea between four and five o’clock was thinner, but there was no sign of perforation. The conjunctival/scleral examination revealed persistent 3+ conjunctival injection, 360-degree chemosis, and one remaining inferotemporal conjunctival nodule. The patient was instructed to continue the use of fortified antibiotics, albeit less frequently, with AST four times daily. The improvement of the corneal ulcer and scleritis was noted to be very slow; fungal culture was taken again but remained negative. At one month following discharge, slit-lamp examination of the left eye showed a healed corneal epithelial defect with infiltrate resolution replaced by scarring without any perforation. The anterior chamber showed an improved view. The conjunctiva and sclera still exhibited 3+ injection, with 360-degree 1+ chemosis and an inferotemporal conjunctival nodule still present. After two months of close follow-ups, the patient was started on topical prednisolone acetate 1% in addition to AST at a frequency of 1 drop every two hours. The patient’s best-corrected vision was CF near face; however, she reported significant improvement in pain and discomfort, with a thin sclera devoid of nodules (Figure 4).

## Discussion

Infectious scleritis is an uncommon cause of scleritis, but when it occurs,* P. aeruginosa *is the pathogen most frequently identified [1]. The histopathological features of *P. aeruginosa* scleritis include extensive ischemic necrosis with microabscesses containing neutrophils and bacteria. This differentiates it from autoimmune or idiopathic scleritis, where microabscesses are absent on pathology [8].* P. aeruginosa* is capable of penetrating beneath an intact conjunctiva into the deep avascular collagen layers of the sclera, where antibiotics struggle to reach therapeutic levels, allowing the bacteria to persist despite aggressive antibiotic treatment [9,10]. Moreover, *P. aeruginosa *can remain in the scleral collagen without provoking an inflammatory response, with recurrence documented in previously uninvolved areas of the eye [9]. Studies reporting the outcomes of infectious scleritis have consistently shown poor results, with 42-88% of affected patients suffering significant vision loss (<20/200). Additionally, despite medical and surgical treatments, 22-33% of cases ultimately require enucleation or evisceration [1]. Several risk factors for PAK have been identified, including advanced age, poor systemic health, adnexal disease, compromised ocular surface, contact lens wear, and the use of topical corticosteroids [11,12]. Additionally, scleritis has been observed following pterygium surgery, particularly when adjunctive treatments like mitomycin C, β-irradiation, or thiotepa were used [11,12]. Although our patient only had advanced age as a risk factor, she presented with advanced PAK, characterized by severe visual acuity impairment. In the literature, PAK has been managed with various treatments, including cryotherapy, conjunctival resection, debridement, intravitreal antibiotics, and antibiotic lavage [1]. While potential complications of scleritis, such as endophthalmitis, retinal detachment, and choroidal detachments, were reported in the literature, they did not occur in our case [1]. Most eyes with *P. aeruginosa *anterior scleritis treated solely with topical antibiotics tend to result in poor outcomes, often leading to globe perforation or leaving the patient with either no light perception (NLP) or only light perception vision (LP) [13]. However, there have been cases where intensive antibiotic therapy alone has successfully managed PAK [9,10,13,14]. Systemic (parenteral) antibiotic treatment can help slow or prevent the progression of scleritis, as the sclera is closely associated with blood vessels in the uveal, episcleral, and emissary regions, allowing better access for the medication [12]. An alternative approach for managing PAK is the subpalpebral lavage technique, which enhances antibiotic penetration into the tissues [15]. This method provides continuous irrigation, effectively flushing out inflammatory debris and exudates, thereby reducing collagenase and cytokine activity and facilitating infection clearance [15]. By minimizing the need for frequent, intensive fortified antibiotic applications, this technique reduces the nursing care burden and addresses compliance issues [15]. Other studies have indicated that combining surgical debridement with appropriate antimicrobial therapy reduced treatment duration and led to better visual outcomes [1]. Additionally, the use of autologous serum tears as adjuvant therapy has been found to be effective in supporting the healing process of corneal ulcers [16-18].

Furthermore, the use of corticosteroids has been reported in the literature; however, conflicting results exist, with the majority of studies concluding no significant difference [19-21]. The American Academy of Ophthalmology suggests that although there may be a role for corticosteroids in the treatment of bacterial corneal ulcers, there is insufficient evidence to make an official recommendation [22]. Additionally, in our case, there was an increased risk of perforation due to corneal thinning, so corticosteroids were not used as an adjuvant therapy early on.

Moreover, a large Korean study looked into 20 patients with infectious scleritis; they noted that their hospitalization duration was 11.3 days, with an average symptom duration of 16.8 days before presentation. They reported that prior pterygium surgery was the most common risk factor [23]. Surgical intervention was performed in 90% of cases with an average of 4.1 days after admission [23]. The study concluded that identifying causative organisms and applying targeted antibiotic therapy, combined with surgery, could enhance visual prognosis [23].

In comparison, our patient had no history of ocular surgery or trauma and was admitted for only four days and presented just four days after symptom onset, allowing for timely intervention. This likely contributed to her successful recovery with medical treatment alone, highlighting the importance of early management in achieving positive outcomes in PAK. Despite the short hospital stay, our patient had a prolonged treatment course, requiring a two-month follow-up, during which we questioned the possibility of a superimposed fungal infection.

## Conclusions

This case underscores the aggressive nature of *P. aeruginosa *eye infections and the critical importance of prompt, effective treatment. It also underscores the need to maintain a low threshold for considering scleritis in the initial presentation of keratitis. Our 75-year-old patient with PAK responded well to intensive antibiotic therapy, including topical and intravenous medications, along with subconjunctival injections. Early presentation and swift, targeted management were key factors for the improvement of the patients’ symptoms and resolution of the infection. Nonetheless, the patient’s final vision remained limited due to the severity of PAK, and a corneal transplant will likely be necessary in the future to enhance her visual prognosis.
